# Supplementary material for: Two-Dimensional ZnS/SnS2 Heterojunction as a Direct Z-Scheme Photocatalyst for Overall Water Splitting: A DFT Study
Source: Materials (Basel). 2022 May 26;15(11):3786. doi: 10.3390/ma15113786 (PMC9181711; doi:10.3390/ma15113786)
Supplement: Supplementary file 1 [file materials-15-03786-s001.zip › materials-1708809-supplementary.pdf]

Supplementary Materials

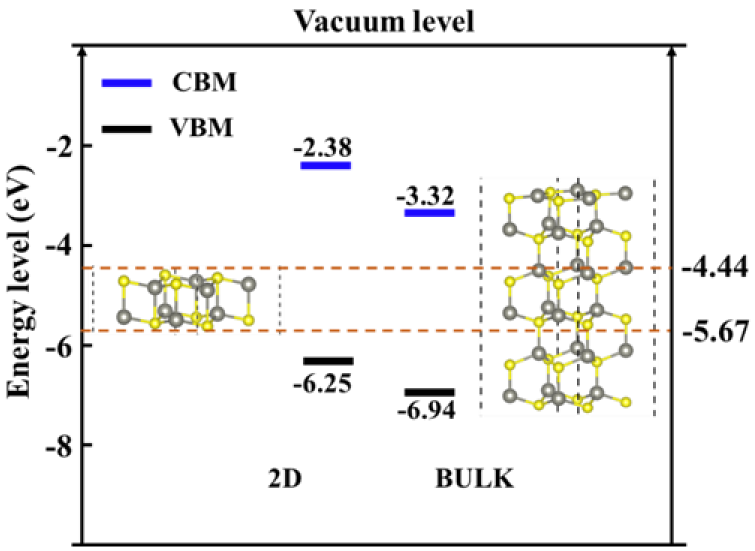

Figure S1. the band edges with respect to the vacuum level of 2D ZnS and bulk ZnS, as well as the reduction (H^+/H_2) and oxidation (O_2/H_2O) potentials at pH=0.
